# *QuickStats:* Percentage* of Children^†^ Aged 3–17 Years Who Ever Received a Diagnosis of Attention-Deficit/Hyperactivity Disorder,^§^ by Sex and Age Group — National Health Interview Survey,^¶^ United States, 2019

**DOI:** 10.15585/mmwr.mm7029a3

**Published:** 2021-07-23

**Figure:**
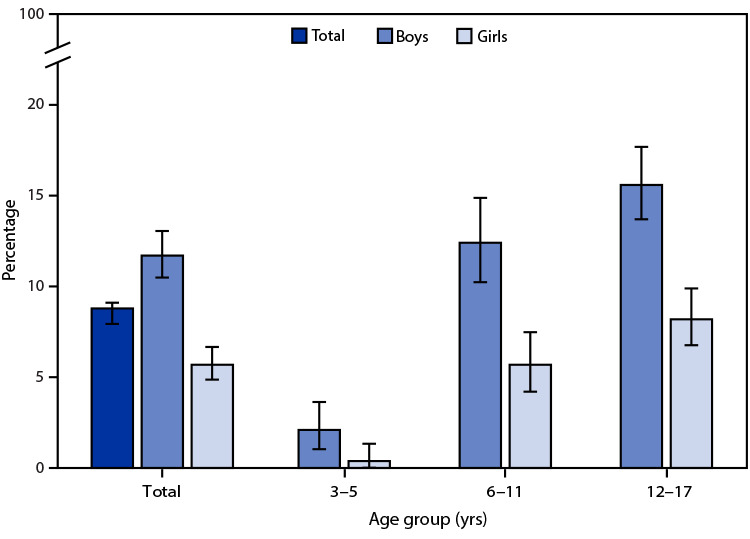
Overall, in 2019, 8.8% of children aged 3–17 years had ever received a diagnosis of ADHD. Boys (11.7%) were more likely than girls (5.7%) to receive a diagnosis of ADHD overall and within each age group. Among both boys and girls, the percentage of children who had ever received a diagnosis of ADHD increased with increasing age group.

**Source:** National Center for Health Statistics, National Health Interview Survey, 2019. https://www.cdc.gov/nchs/nhis/index.htm

